# Potential role of Triggering Receptor Expressed on Myeloid Cells-1 (TREM-1) in SARS-CoV-2 infection: First insights

**DOI:** 10.17179/excli2021-3581

**Published:** 2021-03-26

**Authors:** Ayane de Sá Resende, Yrna Lorena Matos de Oliveira, Tatiana Rodrigues de Moura, Paulo Ricardo Martins-Filho

**Affiliations:** 1 Health Sciences Graduate Program, Federal University of Sergipe, Aracaju, Sergipe, Brazil; 2 Investigative Pathology Laboratory, Federal University of Sergipe, Aracaju, Sergipe, Brazil

## ⁯

***Dear Editor,***

Despite of the ongoing vaccination against Severe Acute Respiratory Syndrome Coronavirus 2 (SARS-CoV-2) infection, the high transmissibility and severity of coronavirus disease (COVID-19) is still a worldwide concern. The management of COVID-19 cases in hospital strongly relies on prognostic markers for supportive care and treatment of complications, although no specific antiviral therapy has been available. In addition, the most studied pharmacologic agents have shown controversial results in critically ill patients with COVID-19, which may progress to Acute Respiratory Distress Syndrome (ARDS), sepsis and multiple organ failure (Martins-Filho et al., 2020[4]). As observed in distinct viral infections, an exacerbated immune response may lead to tissue damage and systemic complications that worsen the prognosis of patients. In this scenario, investigating potential biomarkers for SARS-CoV-2 infection is critical for understanding biological agents and target therapies. 

Triggering Receptor Expressed on Myeloid Cells-1 (TREM-1) is an important member of the TREM family expressed in human myeloid cells. Its activation results in DAP12 signaling, which leads to a substantial production of inflammatory mediators, including TNF-α, IL-6, IL-8 and IL-1β (de Oliveira Matos et al., 2020[2]). The soluble form (sTREM-1), cleaved from the cell membrane, is a practical inflammatory marker that can be measured in human body fluids. The role of sTREM-1 has been extensively evaluated in bacterial infections, especially as a diagnosis and prognosis marker for sepsis and pneumonia (Roe et al., 2014[6]). Furthermore, sTREM-1 was found to be the better biomarker for individuals at risk of all-cause febrile mortality compared to C-reactive protein (CRP) and procalcitonin (Richard-Greenblat et al., 2020[5]). Recently, it has been suggested the potential involvement of TREM-1 signaling in the pathogenesis of several viral infections (Amrun et al., 2020[1]; Ruiz-Pacheco et al., 2014[7]) including COVID-19 (Van Singer et al., 2021[8]). 

Van Singer et al. (2021[8]) have demonstrated that sTREM-1, in combination with respiratory rate, had the best prognostic accuracy for 30-day intubation/mortality in COVID-19 patients compared to CRP with 94 % sensitivity. In addition, severe COVID-19 patients presented significant higher levels of sTREM-1 at the initial phase of infection, thus, highlighting its relevance as useful triage tool to decide on outpatient management. High levels of sTREM-1 could be associated with either a virus-induced compensatory mechanism to counteract inflammatory process, or a host-induced mechanism to control tissue damage by attenuating downstream inflammatory signals (Roe et al., 2014[6]). Moreover, it has been found that sTREM-1 can be an early predictor of 28-day mortality in patients with ARDS (Lin et al., 2010[3]), which may occur as a severe complication in critically ill patients with COVID-19. Despite function of sTREM-1 has not been well established and there is an important gap in understanding the development and resolution of the immune response against SARS-CoV-2 infection, we hypothesized that TREM-1 and its soluble form may play a critical role in the hyperinflammatory response and poor outcomes in patients with COVID-19.

In summary, these first insights suggest that TREM-1 and its soluble form may play a pivotal role in the pathogenesis of SARS-CoV-2 infection. Further studies should investigate the clinical relevance of this inflammatory biomarker in COVID-19. 

## Funding

There is no funding source. 

## Conflict of interest

There is no conflict of interest.
